# Different Manifestations in Familial Isolated Left Ventricular Non-compaction: Two Case Reports and Literature Review

**DOI:** 10.3389/fped.2020.00370

**Published:** 2020-07-07

**Authors:** Hamida Al Hussein, Hussam Al Hussein, Valentin Stroe, Marius Harpa, Claudiu Ghiragosian, Cristina Maria Goia, Carmen Elena Opris, Horatiu Suciu

**Affiliations:** ^1^Department of Morphological Sciences, University of Medicine and Pharmacy of Târgu Mureş, Târgu Mureş, Romania; ^2^Department of Cardiovascular Surgery, University of Medicine and Pharmacy of Târgu Mureş, Târgu Mureş, Romania; ^3^Department of Cardiology, University of Medicine and Pharmacy of Târgu Mureş, Târgu Mureş, Romania

**Keywords:** left ventricular non-compaction, familial, dilated cardiomyopathy, heart transplantation, peripartum cardiomyopathy, ventricular tachycardia

## Abstract

Left ventricular non-compaction (LVNC) is a form of cardiomyopathy characterized by prominent trabeculae and deep intertrabecular recesses which form a distinct “non-compacted” layer in the myocardium. It results from intrauterine arrest of the compaction process of the left ventricular myocardium. Clinical manifestations vary from asymptomatic to heart failure (HF), arrhythmias, or thromboembolic events. We present a case of mother and son diagnosed with isolated LVNC (ILVNC). A 4-years-old male patient, diagnosed at 3 months with ILVNC, and NYHA functional class IV HF, was admitted to the Emergency Institute for Cardiovascular Diseases and Transplantation of Targu Mures, Romania, for cardiologic reevaluation, and diagnosis confirmation. ILVNC was confirmed using echocardiography, revealing a non-compaction to compaction (NC/C) ratio of > 2.7. His evolution was stationary until the age of 8 years, when severe pneumonia caused hemodynamic decompensation, and he was listed for heart transplantation (HT). The patient underwent HT at the age of 11 years with favorable postoperative outcome. Meanwhile, a 22-years-old female patient, mother of the aforementioned patient, was also admitted to our institute due to severe fatigue, dyspnea, and recurrent palpitations with multiple implantable cardioverter defibrillator (ICD) shock delivery. Extensive medical history revealed that a presumptive ILVNC diagnosis was established when she was 11 years old. She was asymptomatic until 18 years old, when 3 months post-partum, she developed NYHA functional class III HF, and subsequently underwent ICD implantation. Her diagnosis was confirmed using multi-detector computed tomography angiography, which revealed a NC/C ratio of > 3.3. ICD adjustments were carried out with a favorable evolution under chronic drug therapy. The last evaluation, at 27 years old, revealed that she was in NYHA functional class II HF. In conclusion, ILVNC, even when familial, can present different clinical pictures and therefore requires different medical approaches.

## Introduction

Left ventricular non-compaction (LVNC) is a rare cardiomyopathy characterized by prominent trabeculae and deep intertrabecular recesses that communicate with the left ventricular (LV) cavity, along with two distinct layers of the ventricular myocardium: compacted and non-compacted (1–4). It results from intrauterine arrest of the compaction process of the LV myocardium (2, 3). It is classified as a primary genetic cardiomyopathy according to American Heart Association (AHA) and an unclassified cardiomyopathy according to the European Society of Cardiology (ESC) (5, 6). The estimated incidence is 0.81/100.000 infants/year and 0.21/100.000 children/year with a prevalence of 0.01–0.26% in adults referred to echocardiography (4, 7, 8). Isolated LVNC (ILVNC) is defined as occurring in the absence of other congenital cardiac malformations (9).

Varying underlying genetic causes lead to a wide range of clinical manifestations. We present two case reports of familial ILVNC with widely different presentations and clinical courses.

## Case Presentations

### Case Report 1

**Figure 1:**
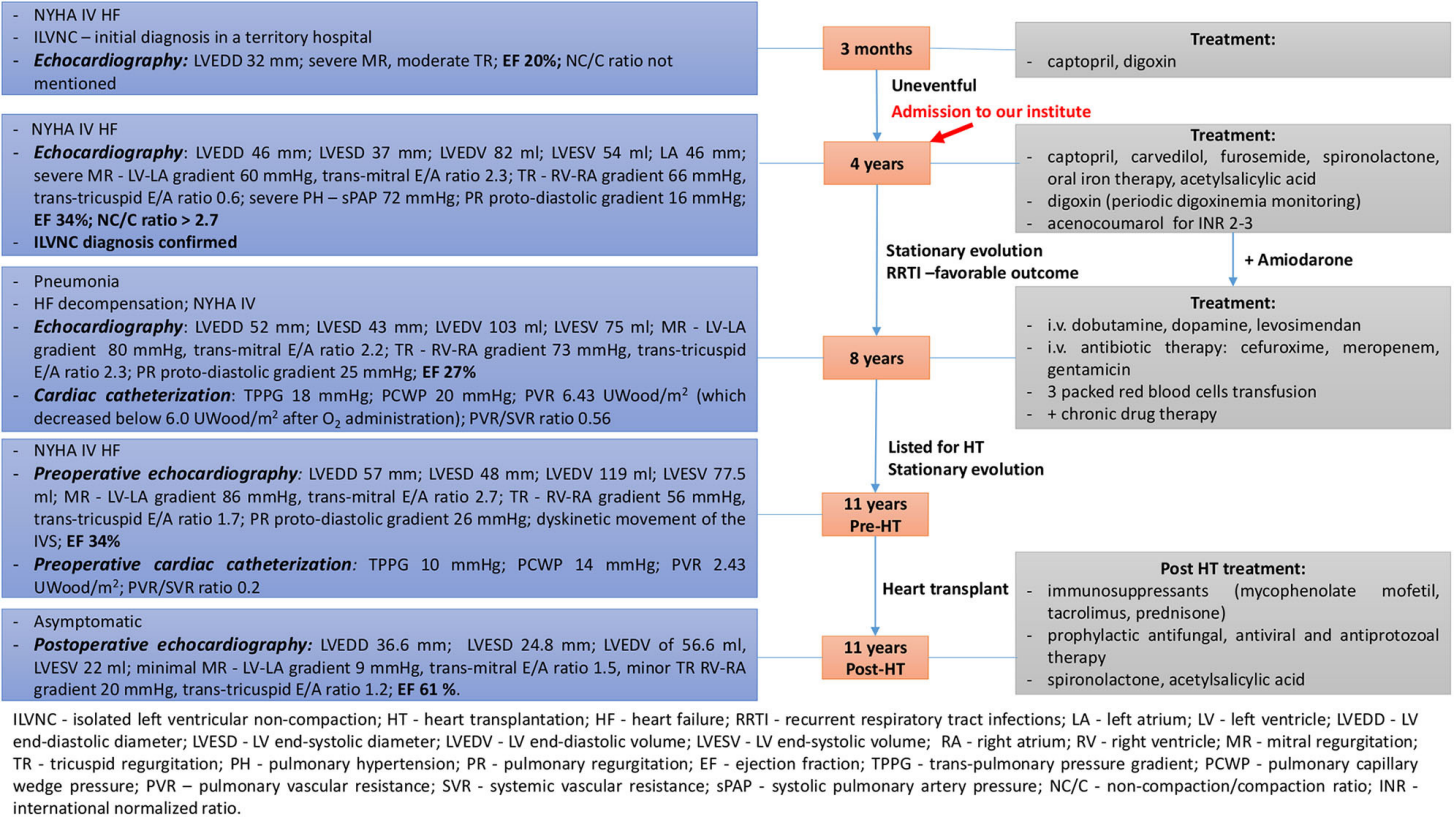
Summative timeline showcasing key findings of the patient's evolution and detailed treatment (case 1).

A 4-years-old male patient, diagnosed at the age of 3 months with ILVNC and NYHA functional class IV heart failure (HF) in a regional hospital, was admitted to our institute; the Emergency Institute for Cardiovascular Diseases and Transplantation of Targu Mures, Romania for cardiologic reevaluation, and diagnosis confirmation. A summative timeline showcasing key findings of the patient's evolution and detailed treatment is presented in Figure 1.

*Family history* revealed that his mother had been diagnosed with ILVNC at the age of 11 years. His maternal grandparents, paternal grandmother, aunts, uncles, nephews, and nieces had no significant medical history. His father and paternal grandfather had arterial hypertension, and he had no siblings.

*Clinical symptoms* included dyspnea and fatigue at rest. *Physical examination* revealed failure to thrive, pallor, mild perioral cyanosis, rhythmic heart sounds, increased intensity of the second heart sound in the pulmonary artery area, a III/6 systolic murmur in the mitral area, blood pressure (BP) of 78/52 mmHg, heart rate (HR) of 120 bpm, and an O_2_ saturation (SpO_2_) of 96%. Neurologic physical examination excluded a neuromuscular condition.

*Laboratory* findings revealed iron deficiency anemia, normal creatine kinase levels as well as liver and kidney parameters.

*Chest X-ray* showed cardiomegaly, enlarged pulmonary arteries and pruning of peripheral pulmonary vessels.

*Electrocardiogram (ECG)* showed sinus rhythm of 120 bpm, biatrial enlargement, and biventricular hypertrophy.

**Figure 2:**
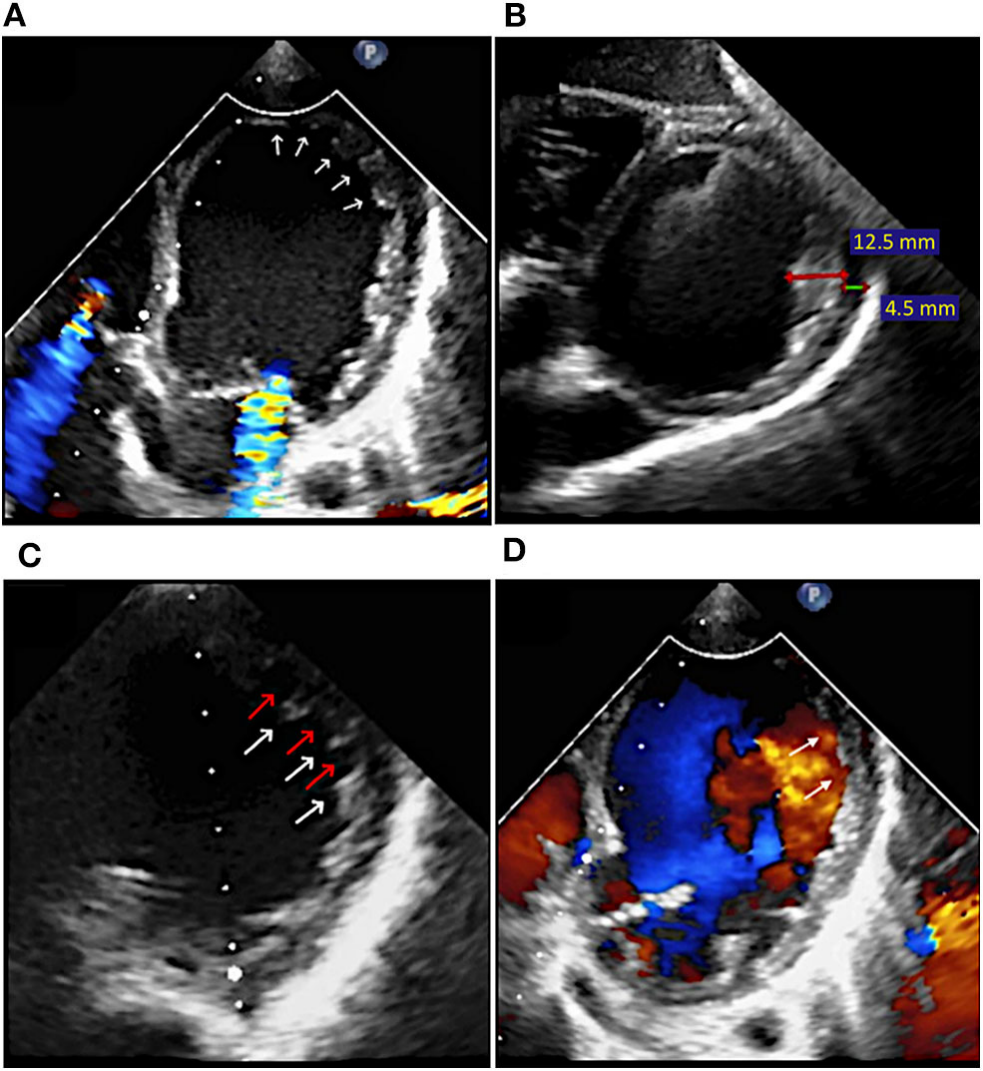
Case 1: Transthoracic echocardiography at the moment of diagnosis: **(A)** Apical and lateral LV trabeculations and intertrabecular recesses (arrows); **(B)** Non-compaction (red line) to compaction (green line) ratio in end-systole of >2.7 (12.5/4.5 mm); **(C)** LV trabeculations (white arrows) and intertrabecular recesses (red arrows) more visible in diastole; **(D)** Color Doppler echocardiography showing direct blood flow from LV cavity into the recesses (arrows) in diastole.

*Echocardiography* revealed dilated left chambers, LA thrombosis and spontaneous echo contrast in the LV. Detailed parameters are shown in Figure 1. A thickened, non-compacted, endocardial layer was observed, with prominent trabeculations and intertrabecular recesses located at the apex and mid-lateral wall of the LV (Figure 2A). The non-compaction to compaction (NC/C) ratio assessed in end-systole was >2.7 (Figure 2B). The trabeculations and intertrabecular recesses were more visible in diastole (Figure 2C). Doppler echocardiography revealed direct blood flow from LV cavity into the recesses (Figure 2D). No other coexisting cardiac abnormalities were observed. These findings were consistent with ILVNC, thus confirming the diagnosis.

The patient's evolution was stationary between ages 4 and 8 years with recurrent respiratory tract infections, but with favorable outcome and no deterioration in the clinical or echocardiographic parameters observed during cardiologic reevaluations. LA thrombosis resolution was observed as well. The patient was following chronic oral drug therapy related in Figure 1. Amiodarone was added subsequently, after the patient related palpitations and ECG Holter monitoring revealed a few isolated, monomorphic premature supraventricular beats.

At the age of 8 years, the patient's status worsened after he developed severe pneumonia. He was admitted with severe expiratory dyspnea and productive cough. Physical examination revealed mild fever, pallor, diaphoresis, tachypnea, intercostal retractions, mild hepatomegaly, BP of 78/50 mmHg, HR of 107 bpm, and SpO_2_ of 93%. Laboratory findings revealed leukocytosis and neutrophilia, slightly elevated C-reactive protein, more severe anemia, and an extremely elevated NT-proBNP of 11,436 pg/ml. Blood and urine cultures were negative. Chest X-ray revealed consolidation of the right lung upper lobe. Echocardiography revealed recurrent LA thrombosis as well as aggravated parameters (Figure 1). Cardiac catheterization was also performed (findings presented in Figure 1). The patient needed intravenous inotropic support, antibiotic therapy, as well as packed red blood cells transfusions for anemia correction (Figure 1), with progressive improvement in the clinical and hemodynamic status. The patient was then listed for heart transplantation (HT) and remained on the waiting list for 3 years, with a stationary evolution, until the age of 11 years old.

At 11 years old, when a donor was available, preoperative echocardiography and cardiac catheterization were performed (Figure 1) and the patient underwent HT with no intraoperative complications and favorable postoperative outcome. Postoperative echocardiography parameters are shown in Figure 1.

### Case Report 2

**Figure 3:**
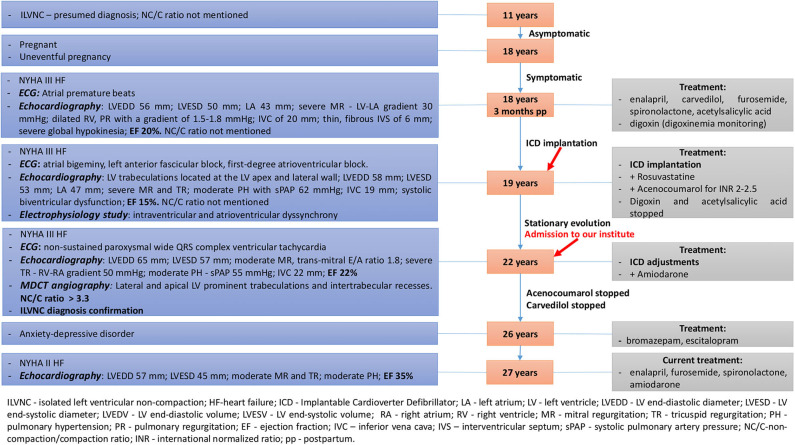
Summative timeline showcasing key findings of the patient's evolution and detailed treatment (case 2).

A 22-years-old female patient, mother of the aforementioned patient was admitted to our institute due to severe fatigue, dyspnea and recurrent palpitations with multiple implantable cardioverter defibrillator (ICD) shock delivery. A summative timeline showcasing key findings of the patient's evolution and detailed treatment is presented in Figure 3.

*Extensive medical history* was conducted by evaluating all previous medical documents revealing the following: At 11 years old, she had a presumptive diagnosis of ILVNC after a general physician detected an apical systolic murmur at routine evaluation and referred her to a cardiologist. She was asymptomatic at that time, until the age of 18 years old. No medications were prescribed and no further cardiologic reevaluations were performed.

At 18 years old, she experienced an uneventful pregnancy. She gave birth through C-section at 37 weeks of gestation to a male infant (case 1) weighing 3,100 g.

Three months post-partum, she was admitted to a regional hospital due to NYHA functional class III HF. She related exertional dyspnea on minimal activity and syncope. Physical examination revealed peripheral edema and ascites. Laboratory findings revealed iron deficiency anemia. ECG showed frequent atrial premature beats. Peripartum cardiomyopathy (PPC) was initially suspected, but echocardiography revealed a non-compaction aspect of the apical and mid-lateral segments of the LV (NC/C ratio was not mentioned in the medical documents). Detailed echocardiographic parameters and drug therapy are mentioned in Figure 3.

At 19, she was readmitted to a regional hospital due to exertional dyspnea on minimal activity, nocturnal paroxysmal dyspnea, dizziness, and syncope. Physical examination showed irregular heart sounds, proto-diastolic gallop, II/6 systolic murmur in the mitral area, and pulmonary basal fine crackles. ECG revealed periods of atrial bigeminy, left anterior fascicular block, and first-degree atrioventricular block. Echocardiography revealed LV trabeculations located at the LV apex and mid-lateral wall (NC/C ratio was not mentioned in the medical documents). Detailed echocardiographic parameters and drug therapy are mentioned in Figure 3. Electrophysiology study revealed intraventricular and atrioventricular dyssynchrony; therefore, the patient underwent ICD implantation. Digoxin was stopped due to its pro-arrhythmic effect, as well as acetylsalicylic acid, as acenocoumarol was added, due to severely decreased ejection fraction (EF), maintaining an INR of 2-2.5. Rosuvastatin was added due to elevated cholesterol levels.

Her evolution was stationary until the age of 22, when she was admitted to our institute.

*Physical examination* at admission revealed altered general condition and a BP of 90/60 mmHg.

*ECG* revealed non-sustained paroxysmal wide QRS complex ventricular tachycardia.

*Echocardiography* parameters are related in Figure 3.

**Figure 4:**
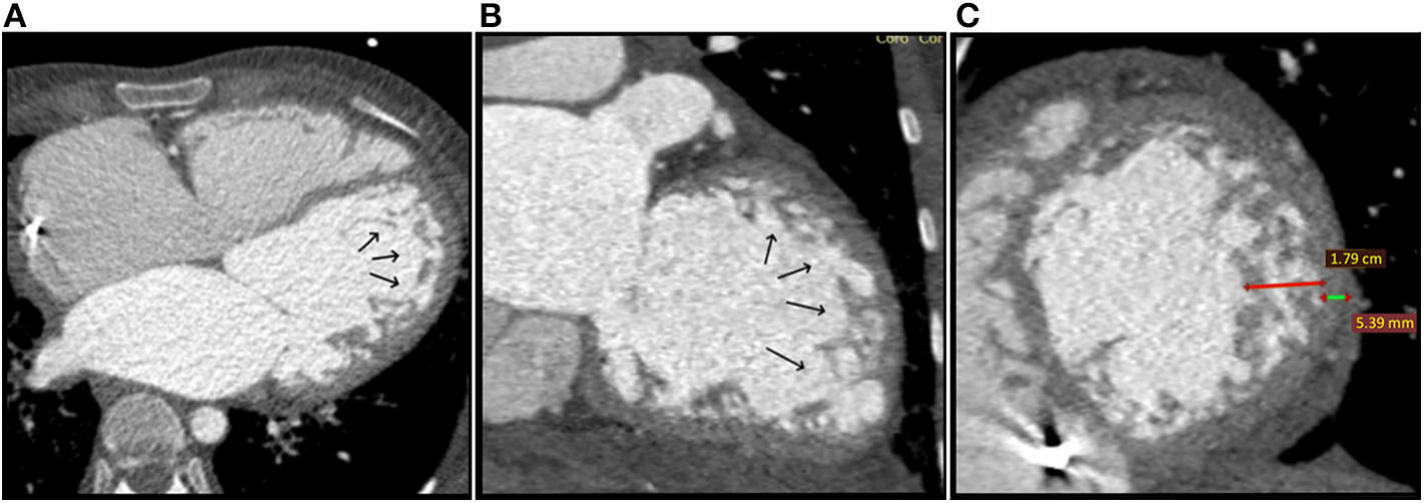
Case 2: Multi-detector Computed Tomography (MDCT) angiography at the moment of diagosis: **(A)** Axial reconstruction and **(B)** Coronal reconstruction: Black arrows show prominent trabeculations and deep intertrabecular recesses located at the apex and lateral wall of the LV; **(C)** Sagital reconstruction, short axis plane showing non-compaction (red line) to compaction (green line) ratio of > 3.3 (17,9/5,39 mm).

*Multi-detector computed tomography (MDCT) angiography* was performed for ILVNC confirmation, as she had an ICD and cardiac magnetic resonance (CMR) was contraindicated, simultaneously exploring the coronary arteries. Mid-lateral and apical LV prominent trabeculations and intertrabecular recesses were observed (Figures 4A,B). The NC/C ratio was >3.3 (Figure 4C). Therefore, the diagnosis was confirmed. Coronary arteries exhibited no significant lesions. ICD malfunction was suspected, and programming adjustments were performed. Amiodarone was added to her oral chronic drug therapy with favorable outcome.

Her evolution was favorable at the following reevaluations, with no more recurrent tachycardia episodes or multiple ICD shocks related, with gradual improvement in EF and NYHA functional class. Oral anticoagulant therapy was stopped as the EF improved (>30%), as well as carvedilol, due to bradycardia. At 26 years old, due to anxiety-depressive disorder, she was prescribed bromazepam and escitalopram by a psychiatrist. During her last cardiologic evaluation, at 27 years old, she was in NYHA functional class II HF and compliant with her cardiologic and psychiatric treatment. Echocardiography parameters are shown in Figure 3.

## Discussion

The underlying genetic causes and the clinical phenotype of LVNC are heterogeneous, therefore, it has been suggested that describing LVNC along with the cardiomyopathy phenotype might be clinically useful: LVNC-dilated phenotype (LVNC-DCM), LVNC-hypertrophic phenotype (LVNC-HCM), LVNC-restrictive phenotype (LVNC-RCM), LVNC- arrhythmogenic phenotype (LVNC-ARVC), and LVNC with normal LV size and function (1, 7, 10).

### Clinical Presentation

Clinical manifestations exist on a wide spectrum, from asymptomatic to the classic triad of HF, arrhythmias, or thromboembolic events. HF is characterized by a systo-diastolic dysfunction (2). Although HF is the most frequent complication of ILVNC both in pediatric and adult populations (11), children seem to be more frequently asymptomatic at presentation, despite the common finding of decreased EF (12), while clinically overt symptoms of HF were more frequent in the adult population (13). In contrast, in our case report, the child presented clinically overt symptoms of HF at a young age, while the onset of HF in the mother was observed later, in adulthood, probably triggered by pregnancy.

ECGs are usually abnormal in patients with LVNC (2) although arrhythmias seem to be slightly less common in pediatric population (14). Supraventricular arrhythmias, ventricular arrhythmias, ranging from isolated uniform ectopic beats to sustained ventricular tachycardia and ventricular fibrillation, as well as various conduction disturbances have been described in patients with LVNC (2, 11, 15–18). Despite the wide range of arrhythmias, ventricular tachycardia (VT) is considered to be the hallmark of LVNC (16). Although VT is more commonly seen in children (2), in our case report, recurrent VT was observed only in the mother. The reported incidence of sudden cardiac death (SCD) ranges from 6.2% in pediatric population (19) to 40 or 50% in the adult population (13, 20). This suggests the possible importance of primary or secondary prevention by ICD implantation. There are no specific guidelines for primary prevention in patients with LVNC; however, current guidelines regarding other types of cardiomyopathies can be extrapolated depending on the phenotype of LVNC (21). Our patient was a candidate for ICD implantation due to recurrent syncope and severe systolic dysfunction. Antiarrhythmic drug therapy proved to be effective in reducing ICD shocks.

Thromboembolic events are the least common manifestation and appear secondary to depressed LV function along with blood stasis within the intertrabecular recesses (2, 16, 17, 22). They seem to be less frequent in children (14). Interestingly, in our case, the child developed recurrent thrombosis despite anticoagulation therapy, while the mother received anticoagulation therapy only for a limited period, due to very decreased EF, without any thrombotic events in her evolution whatsoever. There are no specific guidelines regarding anticoagulation therapy in ILVNC; however, it is justified in the setting of associated systolic dysfunction/atrial fibrillation or thromboembolism history, including intracardiac thrombi (23–26).

### Definitive and Differential Diagnosis

Transthoracic echocardiography is usually the first method used for diagnosing ILVNC (27). The most commonly used set of echocardiographic criteria are those described by Jenni et al. (28), which were met by our patient. If inconclusive, CMR, computed tomography (CT), or contrast ventriculography can be used (2). CMR offers a high diagnostic accuracy (29) and holds an important role in excluding other pathologies (27). However, in the presence of cardiac implantable devices, CMR cannot be performed and CT angiography might be a good alternative (29). As our patient had an ICD, we chose to confirm the diagnosis using MDCT angiography, simultaneously exploring the coronary arteries. The visualization of the trabeculations and intertrabecular recesses was very satisfactory and the NC/C ratio was consistent with ILVNC. Interestingly, the pattern and aspect of the trabeculations and recesses on the mother's MDCT angiography highly resembled those observed on the child's echocardiography, suggesting similar phenotypes. A few case reports were published regarding efficient visualization and diagnosis of ILVNC using CT angiography (27, 29–31). As we acquired accurate and efficient results as well, we consider that MDCT angiography can be a very reliable alternative method for diagnosing ILVNC when MRI cannot be performed.

Differential diagnosis is usually made with other cardiomyopathies, apical hypertrophic cardiomyopathy, aberrant bands, abnormal chords, endocardial fibroelastosis, and abnormal insertion of papillary muscle (27, 32). Towbin et al. described different heterogeneous echocardiographic phenotypes of ILVNC, which can appear very different on echocardiography (33). The differential diagnosis with DCM in an ILVNC with DCM phenotype can be difficult; nevertheless, the criteria of ILVNC are still fulfilled (32). In our case, the echocardiographic diagnosis of the child was particularly challenging, as the highly dilated LV “masked” the trabeculations and intertrabecular recesses, which did not look as prominent as they do in a “typical” non-DCM ILVNC; however, diagnostic criteria of ILVNC were still present, assessed by careful measuring and Doppler echocardiography. Therefore, very detailed echocardiographic examination is required to differentiate between DCM and ILVNC-DCM.

The main differential diagnosis of women with ILVNC in the setting of pregnancy/postpartum period should be performed with PPC. A few cases have been reported regarding pregnant women diagnosed with PPC who simultaneously presented characteristic features of LVNC (34–37). Out of a total of eight patients, none presented complete normalization of LV function at 6 months postpartum. Interestingly, three of these patients had a history of previous uneventful pregnancies. However, a prospective longitudinal study reported development of *de novo* LV trabeculations in primigravida pregnant women with a normal LV size, morphology, and function antepartum. These women did not present LV systolic or diastolic dysfunction whatsoever, and the majority showed complete regression of LV trabeculations within 1 year. These *de novo* LV trabeculations are thought to be an adaptive process in situations with increased cardiac preload, as seen in pregnancy, and have also been reported in athletes and sickle cell anemia patients (38).

As more than 50% of women with PPC recover LV function (39, 40), the patients reported in the aforementioned studies who did not fully recover postpartum, especially given the fact that there were no antepartum echocardiographic data, might have had latent, asymptomatic LVNC with deterioration in the LV function due to pregnancy. In our case report, the mother showed similar clinical manifestations; however, she was known to have ILVNC since 11 years old, which demonstrates that the decompensation of LVNC can mimic PPC. Although she did show improvement in the EF over a 6-years period, a full normalization was not observed, and given the progressive natural history of the disease, a possible future need for HT cannot be excluded. Extensive medical history, clinical, and imaging data, and follow-up reevaluations should be performed to exclude PPC. Many asymptomatic LVNC patients can be overlooked, which may lead to diagnostic confusion and mismanagement of these patients, since the prognosis of LVNC is worse than PPC.

### Genetic Aspects

LVNC can be genetically sporadic or familial. Autosomal-dominant inheritance is most common, followed by X-Linked inheritance (32, 41). Autosomal recessive inheritance has also been observed (42). The first gene found to be responsible for LVNC was G4.5 *(TAZ)*, which is located in the Xq28 chromosome region. It results in severe infantile X-linked cardiomyopathies, including Barth syndrome (characterized by cardiomyopathy, skeletal myopathy, and neutropenia) (32, 33). In our case report, the onset of severe cardiomyopathy in the child at a young age and the mom-to-male child association resembled an X-linked inheritance, however, unlike our case 2, female carriers of this mutation are clinically healthy and show no features of ILVNC or reduced systolic function on echocardiography (43, 44). Moreover, the child presented no evidence of neuromuscular condition whatsoever.

Lamin A/C *(LMNA)* is another gene responsible for the development of LVNC and DCM (2). This shows the genetic overlap of different cardiomyopathies (45) and might explain the severe DCM phenotype in some patients with ILVNC (25).

Mutations of sarcomere protein genes, such as beta-myosin heavy chain *(MYH7)*, alfa cardiac actin *(ACTC)*, and cardiac troponin T *(TNNT2)*, known to cause DCM and HCM, can also cause LVNC (2, 32) again, showing a genetic overlap of different cardiomyopathies. A significant number of patients exhibiting *MYH7* mutation can be clinically asymptomatic but with echocardiographic criteria of ILVNC, while members of the same family can exhibit severe symptomatic ILVNC with HF (46). This might explain the discrepancy between the clinical course and symptom onset in our patients.

Mutations in alpha-dystrobrevin (1), *ZASP* (Z-band alternatively spliced PDZ-motif protein) (2, 32) as well as ion channel related genes can cause LVNC as well (1, 2, 41).

This genetic heterogeneity explains both the genotypic overlap of different cardiomyopathies, as well as the phenotypic overlap that might occur. There are very few case reports regarding pregnant women, known or subsequently diagnosed with ILVNC, who gave birth to children with ILVNC, but no genetic analyses were available (35, 47, 48). As genetic testing was not available in our case report either, we conclude that despite the existence of some clinical manifestation patterns according to the genetic mutation, given the broad genetic variability, in the absence of molecular genetic analysis, only clinical assumptions can be made regarding the causative mutation.

LVNC with DCM phenotype appears to be associated with a higher risk of mortality than other forms of LVNC (10). The treatment guidelines for DCM have proven effective in LVNC-DCM (10). LVNC constitutes a rare indication for heart transplantation in pediatric population (22, 49), but in cases of refractory HF, this might be the only option (8). In Romania, this was the first pediatric HT performed for ILVNC. Our familial case highlights both sides of the spectrum regarding long term management methods and outcome. The mother responded to drug and interventional therapeutic methods, with a favorable evolution, while the child presented refractory HF that required HT at a young age.

ILVNC is a heterogeneous cardiomyopathy with a wide spectrum of clinical manifestations which dictate the subsequent therapeutic approach. While drug therapy can be efficient in some patients, HT can be the only management option for patients with refractory HF. ILVNC can be unpredictable during pregnancy and requires rigorous monitoring as well as further studies to establish certain guidelines and determine whether pregnancy is contraindicated.

## Data Availability Statement

All datasets generated for this study are included in the article/supplementary material.

## Ethics Statement

The studies involving human participants were reviewed and approved by Local Committee of Ethics and Scientific Research of the Emergency Institute for Cardiovascular Diseases and Transplantation, Târgu Mureş, Romania. Written informed consent to participate in this study was provided by the participants' legal guardian/next of kin. Written informed consent was obtained from the individual(s), and minor(s)' legal guardian/next of kin, for the publication of any potentially identifiable images or data included in this article. Written informed consent was obtained from the patient's mother (legal guardian) for herself and her child for the publication of this case report. Both patients related adherence to treatment and good quality of life.

## Author Contributions

HaA and HuA conceptualized and designed the study, contributed to data collection and processing, drafted the initial manuscript, reviewed and revised the manuscript. MH, CG, and HuA carried out the donor heart harvesting, designed, and realized data collection. HS, VS, and CMG were involved in surgical management, coordinated and supervised data collection, and critically reviewed the manuscript. CO contributed to procedure planning, patient management, data collection, and processing. All authors approved the final manuscript as submitted and agree to be accountable for all aspects of the work.

## Conflict of Interest

The authors declare that the research was conducted in the absence of any commercial or financial relationships that could be construed as a potential conflict of interest.

## Abbreviations

ILVNC: isolated left ventricular non-compaction
HT: heart transplantation
ICD: Implantable Cardioverter Defibrillator
DCM: dilated cardiomyopathy
HCM: hypertrophic cardiomyopathy
SCD: sudden cardiac death
PPC: peripartum cardiomyopathy
LV: left ventricle
LA: left atrium
ARVC: arrhythmogenic right ventricular cardiomyopathy
LVNC-DCM: LVNC-dilated phenotype
LVNC-HCM: LVNC-hypertrophic phenotype
LVNC-RCM: LVNC-restrictive phenotype
LVNC-ARVC: LVNC-arrythmogenic phenotype
ECG: electrocardiography
CMR: cardiac magnetic resonance
MDCT: Multi-detector Computed Tomography
MR: mitral regurgitation
NC/C: non-compaction to compaction ratio
SpO_2_: oxygen saturation
BP: blood pressure
HR: heart rate
EF: ejection fraction.
